# Tris(cyclo­hexyl­ammonium) *cis*-di­chlorido­bis­(oxalato-κ^2^ *O* ^1^,*O* ^2^)stann­ate(IV) chloride monohydrate

**DOI:** 10.1107/S1600536813026901

**Published:** 2013-10-05

**Authors:** Modou Sarr, Waly Diallo, Aminata Diasse-Sarr, Laurent Plasseraud, Hélène Cattey

**Affiliations:** a Laboratoire de Chimie Minérale et Analytique (LACHIMIA), Département de Chimie, Faculté des Sciences et Techniques, Université Cheikh Anta Diop, Dakar, Senegal; b ICMUB UMR 6302, Université de Bourgogne, Faculté des Sciences, 9 avenue Alain Savary, 21000 Dijon, France

## Abstract

The crystal structure of the title compound, (C_6_H_14_N)_3_[Sn(C_2_O_4_)_2_Cl_2_]Cl·H_2_O, contains three cyclo­hexyl­ammonium cations, one stannate(IV) dianion, one isolated chloride anion and one lattice water mol­ecule. The cyclo­hexyl­ammonium cations adopt chair conformations. In the complex anion, two bidentate oxalate ligands and two chloride anions in *cis* positions coordinate octa­hedrally to the central Sn^IV^ atom. The cohesion of the mol­ecular entities is ensured by the formation of N—H⋯O, O—H⋯O, O—H⋯Cl and N—H⋯Cl inter­actions involving cations, anions and the lattice water mol­ecule, giving rise to a layer-like arrangement parallel to (010).

## Related literature

For general background on organotin(IV) chemistry and applications, see: Evans & Karpel (1985 ▶); Davies *et al.* (2008 ▶). For previous studies of tin(IV) derivatives with oxidoanions, see: Sarr & Diop (1990 ▶); Qamar-Kane & Diop (2010 ▶); Diallo *et al.* (2009 ▶). For crystal structures of halogenidotin(IV) compounds, see: Willey *et al.* (1998 ▶); Skapski *et al.* (1974 ▶); Gueye *et al.* (2011 ▶); Sow *et al.* (2013 ▶); Sarr *et al.* (2013 ▶).
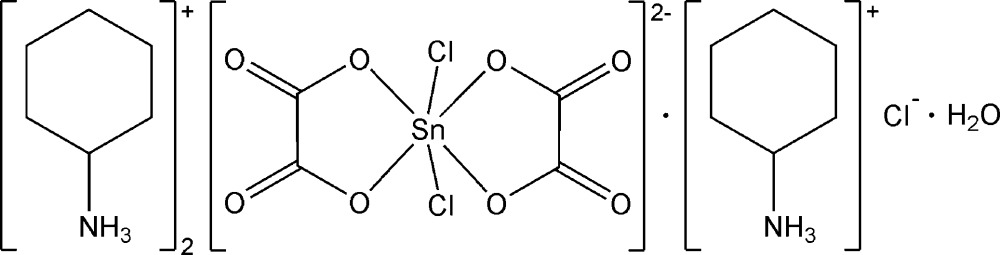


## Experimental

### 

#### Crystal data

(C_6_H_14_N)_3_[Sn(C_2_O_4_)_2_Cl_2_]Cl·H_2_O
*M* *_r_* = 719.64
Monoclinic, 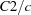

*a* = 27.9894 (10) Å
*b* = 12.3088 (5) Å
*c* = 19.3457 (7) Å
β = 105.542 (1)°
*V* = 6421.2 (4) Å^3^
*Z* = 8
Mo *K*α radiation
μ = 1.09 mm^−1^
*T* = 115 K
0.17 × 0.08 × 0.03 mm

#### Data collection

Nonius KappaCCD diffractometer
10624 measured reflections
7264 independent reflections
6028 reflections with *I* > 2σ(*I*)
*R*_int_ = 0.028

#### Refinement

*R*[*F* ^2^ > 2σ(*F* ^2^)] = 0.049
*wR*(*F* ^2^) = 0.095
*S* = 1.22
7264 reflections
346 parameters
H-atom parameters constrained
Δρ_max_ = 0.66 e Å^−3^
Δρ_min_ = −0.70 e Å^−3^

### 

Data collection: *COLLECT* (Nonius, 1998 ▶); cell refinement: *DENZO-SMN* (Otwinowski & Minor, 1997 ▶); data reduction: *DENZO-SMN*; program(s) used to solve structure: *SIR92* (Altomare *et al.*, 1993 ▶); program(s) used to refine structure: *SHELXL97* (Sheldrick, 2008 ▶); molecular graphics: *ORTEP-3* (Farrugia, 2012 ▶) and *Mercury* (Macrae *et al.*, 2008 ▶); software used to prepare material for publication: *WinGX* (Farrugia, 2012 ▶).

## Supplementary Material

Crystal structure: contains datablock(s) global, I. DOI: 10.1107/S1600536813026901/wm2771sup1.cif

Structure factors: contains datablock(s) I. DOI: 10.1107/S1600536813026901/wm2771Isup2.hkl

Supplementary material file. DOI: 10.1107/S1600536813026901/wm2771Isup3.cdx

Additional supplementary materials:  crystallographic information; 3D view; checkCIF report

## Figures and Tables

**Table 1:** Hydrogen-bond geometry (Å, °)

*D*—H⋯*A*	*D*—H	H⋯*A*	*D*⋯*A*	*D*—H⋯*A*
N1—H1*A*⋯O4^i^	0.89	2.11	2.957 (4)	160
N1—H1*B*⋯Cl3^i^	0.89	2.29	3.163 (4)	166
N1—H1*C*⋯O8	0.89	2.05	2.873 (4)	154
N1—H1*C*⋯O7	0.89	2.50	3.130 (5)	129
N2—H2*A*⋯O4^ii^	0.89	1.99	2.829 (4)	157
N2—H2*A*⋯O3^ii^	0.89	2.56	3.197 (4)	129
N2—H2*B*⋯Cl3^i^	0.89	2.41	3.209 (3)	150
N2—H2*C*⋯O6^iii^	0.89	2.00	2.879 (4)	170
N3—H3*A*⋯Cl3	0.89	2.37	3.180 (3)	152
N3—H3*A*⋯O7	0.89	2.48	2.971 (4)	115
N3—H3*B*⋯O9	0.89	1.88	2.751 (5)	164
N3—H3*C*⋯O1^iv^	0.89	2.08	2.957 (4)	167
O9—H1*O*⋯Cl3^i^	0.90	2.21	3.108 (3)	173
O9—H2*O*⋯O3^iv^	0.87	2.28	2.950 (4)	135

Symmetry codes: (i) 
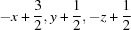
; (ii) 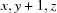; (iii) 
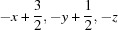
; (iv) 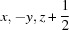.

**Fig. 1.**
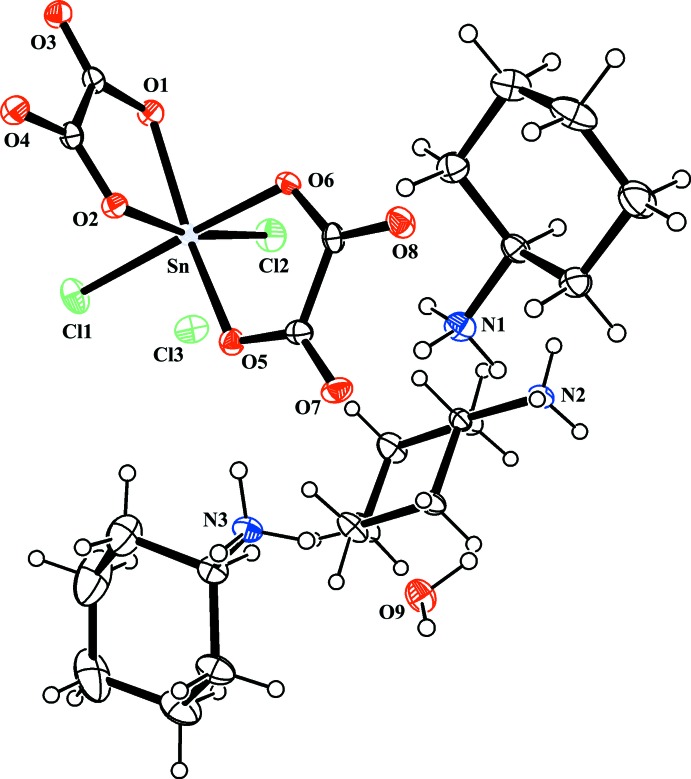
The molecular structure of the title compound with partial atom labelling. Colour code: Sn light grey, O red, N blue, Cl green. Displacement ellipsoids are draw at the 30% probability level.

**Fig. 2.**
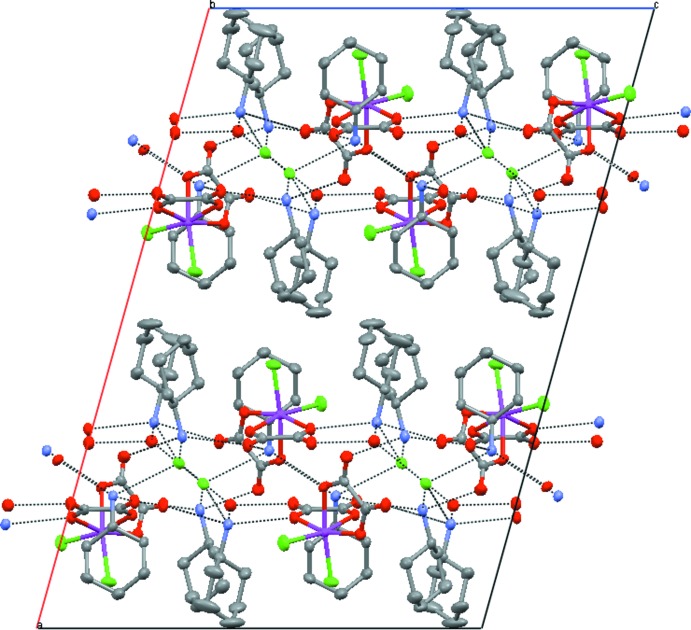
The crystal packing of the title compound viewed along the b axis, showing the layer-like arrangement through intermolecular hydrogen bonding interactions N—H···O; O—H···Cl (dashed lines). Hydrogen atoms are omitted for clarity. Colour code: Sn pink, O red, N blue, Cl green, C grey.

## supplementary crystallographic information

### 1. Comment

The interest to synthesize new organotin derivatives is related to their applications in numerous fields like agrochemicals, catalysis, medicine, surface disinfectants and marine antifouling paints (Evans & Karpel, 1985; Davies *et al.*, 2008). Our group is involved from a long time in the synthetic quest of new organotin compounds, focusing in particular on the coordination affinity with oxoanions (Sarr & Diop, 1990; Diallo *et al.*, 2009; Qamar-Kane & Diop, 2010; Gueye *et al.*, 2011; Sow *et al.*, 2013; Sarr *et al.*, 2013). Thus, in the course of our ongoing studies on oxalato tin(IV) derivatives, we report herein the structure determination of the reaction product (C_6_H_14_N)_3_[Sn(C_2_O_4_)_2_Cl_2_]Cl^.^H_2_O obtained from the reaction between [(C_6_H_14_N)]_2_[C_2_O_4_]^.^1.5H_2_O and SnCl_2_^.^2H_2_O. To the best of our knowledge, this is the first crystallographic report of a compound containing a [dihalogenido-bis(oxalato)stannate(IV)] anion.

The molecular entities of the title structure are shown in Fig. 1. The Sn(IV) atom of the stannate anion is six-coordinated by four oxalate oxygen atoms and two terminal chlorido anions in *cis*-position in a distorted octahedral geometry [Cl1–Sn–Cl2 = 97.37 (4)°, O1–Sn–O2 = 78.19 (10)°, O5–Sn–O6 = 79.99 (10)°]. The bidentate oxalato ligands are nearly planar with O1—C1—C2—O2 and O5—C3—C4—O6 torsion angles of 1.1 (6) and 2.7 (5)°, respectively. They form a dihedral angle of 86.62 (17)° between each other. The Sn—Cl distances [Sn–Cl1 = 2.3370 (11) Å, Sn–Cl2 = 2.3466 (10) Å] as well as the Sn–O distances [Sn–O1 = 2.097 (3) Å, Sn–O2 = 2.098 (3) Å, Sn–O5 = 2.060 (3) Å, Sn–O6 = 2.097 (3) Å] are in the typical range of Sn—Cl and Sn—O bonds reported previously in the literature (Willey *et al.*, 1998; Skapski *et al.*, 1974; Sow *et al.*, 2013). The charges of the [Sn(C_2_O_4_)_2_Cl_2_]^2-^ dianion and the isolated Cl^-^ anion are compensated by three [(C_6_H_11_NH_3_)]^+^ cations, all of which adopt in chair conformations. One uncoordinating water molecule is also present in the crystal lattice.

From a supramolecular view, three of the four oxygen atoms of each oxalato ligand are involved in hydrogen bonging interactions with the lattice water molecule and the surrounding cyclohexylammonium cations through O—H···O and N—H···O contacts, respectively (Table 1). The lattice water molecule is also involved in short contacts with the neighboring isolated Cl^-^ anion and a [(C_6_H_11_NH_3_)]^+^ cation through O—H···Cl and N—H···O contacts, respectively. The isolated Cl^-^ anion is additionally hydrogen-bonded to the three cations through N—H···Cl interactions. The supramolecular contributions lead to the formation of layers extending parallel to (010) as shown in Fig. 2.

### 2. Experimental

All chemicals were purchased from Sigma-Aldrich or Merck and used without further purification. Crystals of the title compound were obtained by reacting [(C_6_H_14_N)]_2_[C_2_O_4_]^.^1.5H_2_O (0.14 g, 0.44 mmol) with SnCl_2_^.^2H_2_O (0.2 g, 0.88 mmol) in 75 ml of ethanol (96% purity) in an 1:2 molar ratio. The mixture was stirred during several hours at room temperature. Slow solvent evaporation yielded colorless crystals suitable for an X-ray crystallographic study.

### 3. Refinement

All H atoms, on carbon and nitrogen atoms, were placed at calculated positions using a riding model with C—H = 0.97 Å (methylene) or 0.98 Å (methine) or N—H = 0.89 Å (amine) with *U*_iso_(H) = 1.2*U*_eq_(C) or *U*_iso_(H) = 1.5*U*_eq_(N). H atoms on water molecule were located in Fourier difference maps and were refined using a riding model with *U*_iso_(H) = 1.2*U*_eq_(O).

### Crystal data

**Table:**

(C_6_H_14_N)_3_[Sn(C_2_O_4_)_2_Cl_2_]Cl·H_2_O	*F*(000) = 2960
*M**_r_* = 719.64	*D*_x_ = 1.489 Mg m^−^^3^
Monoclinic, *C*2/*c*	Mo *K*α radiation, λ = 0.71073 Å
Hall symbol: -C 2yc	Cell parameters from 59056 reflections
*a* = 27.9894 (10) Å	θ = 1.0–27.5°
*b* = 12.3088 (5) Å	µ = 1.09 mm^−^^1^
*c* = 19.3457 (7) Å	*T* = 115 K
β = 105.542 (1)°	Prism, colourless
*V* = 6421.2 (4) Å^3^	0.17 × 0.08 × 0.03 mm
*Z* = 8	

### Data collection

**Table:**

Nonius KappaCCD diffractometer	6028 reflections with *I* > 2σ(*I*)
Radiation source: fine-focus sealed tube	*R*_int_ = 0.028
Graphite monochromator	θ_max_ = 27.5°, θ_min_ = 3.0°
φ scans (κ = 0) + additional ω scans	*h* = −36→36
10624 measured reflections	*k* = −15→10
7264 independent reflections	*l* = −25→25

### Refinement

**Table:**

Refinement on *F*^2^	Primary atom site location: structure-invariant direct methods
Least-squares matrix: full	Secondary atom site location: difference Fourier map
*R*[*F*^2^ > 2σ(*F*^2^)] = 0.049	Hydrogen site location: inferred from neighbouring sites
*wR*(*F*^2^) = 0.095	H-atom parameters constrained
*S* = 1.22	*w* = 1/[σ^2^(*F*_o_^2^) + 39.7649*P*] where *P* = (*F*_o_^2^ + 2*F*_c_^2^)/3
7264 reflections	(Δ/σ)_max_ = 0.003
346 parameters	Δρ_max_ = 0.66 e Å^−^^3^
0 restraints	Δρ_min_ = −0.70 e Å^−^^3^

### Special details

**Table:**

Geometry. All e.s.d.'s (except the e.s.d. in the dihedral angle between two l.s. planes) are estimated using the full covariance matrix. The cell e.s.d.'s are taken into account individually in the estimation of e.s.d.'s in distances, angles and torsion angles; correlations between e.s.d.'s in cell parameters are only used when they are defined by crystal symmetry. An approximate (isotropic) treatment of cell e.s.d.'s is used for estimating e.s.d.'s involving l.s. planes.
Refinement. Intensities at low angles are poorly measured and three reflections with Error/e.s.d. greater than 4 have been omitted for convenience (respectively, 4.86, 4.84 and 4.24).

### Fractional atomic coordinates and isotropic or equivalent isotropic displacement parameters (Å^2^)

**Table:**

	*x*	*y*	*z*	*U*_iso_*/*U*_eq_	
Sn	0.84481 (2)	−0.01264 (2)	0.08670 (2)	0.02266 (7)	
O1	0.82137 (11)	−0.1343 (2)	0.00914 (14)	0.0275 (6)	
O2	0.81693 (10)	−0.1304 (2)	0.14399 (14)	0.0247 (6)	
O3	0.79899 (11)	−0.3092 (2)	−0.00137 (15)	0.0313 (6)	
O4	0.79534 (11)	−0.3041 (2)	0.14046 (14)	0.0307 (6)	
O5	0.84818 (10)	0.0972 (2)	0.16821 (14)	0.0277 (6)	
O6	0.77157 (10)	0.0445 (2)	0.05761 (14)	0.0273 (6)	
O8	0.72353 (11)	0.1682 (3)	0.08902 (16)	0.0366 (7)	
O7	0.80129 (12)	0.2174 (2)	0.20695 (15)	0.0332 (7)	
Cl1	0.92508 (4)	−0.08025 (10)	0.13280 (7)	0.0418 (3)	
Cl2	0.86238 (5)	0.10594 (10)	0.00174 (6)	0.0429 (3)	
C3	0.80701 (16)	0.1493 (3)	0.1644 (2)	0.0265 (8)	
C4	0.76247 (15)	0.1196 (3)	0.0982 (2)	0.0256 (8)	
C1	0.80865 (15)	−0.2263 (3)	0.0328 (2)	0.0244 (8)	
C2	0.80675 (15)	−0.2225 (3)	0.1126 (2)	0.0240 (8)	
N1	0.69051 (13)	0.2526 (3)	0.20683 (17)	0.0297 (8)	
H1A	0.6955	0.2190	0.2489	0.045*	
H1B	0.6989	0.3222	0.2140	0.045*	
H1C	0.7090	0.2214	0.1815	0.045*	
C5	0.63681 (15)	0.2442 (3)	0.1665 (2)	0.0273 (8)	
H5	0.6318	0.2849	0.1215	0.033*	
C6	0.62238 (16)	0.1271 (3)	0.1480 (2)	0.0331 (9)	
H6A	0.6292	0.0841	0.1916	0.040*	
H6B	0.6419	0.0980	0.1178	0.040*	
C7	0.56753 (18)	0.1192 (4)	0.1089 (3)	0.0451 (12)	
H7A	0.5616	0.1548	0.0627	0.054*	
H7B	0.5584	0.0433	0.1005	0.054*	
C8	0.53516 (18)	0.1710 (5)	0.1512 (3)	0.0493 (13)	
H8A	0.5008	0.1685	0.1232	0.059*	
H8B	0.5380	0.1303	0.1951	0.059*	
C9	0.55019 (18)	0.2879 (4)	0.1696 (3)	0.0478 (13)	
H9A	0.5303	0.3179	0.1991	0.057*	
H9B	0.5440	0.3303	0.1258	0.057*	
C10	0.60496 (16)	0.2953 (4)	0.2098 (2)	0.0346 (10)	
H10A	0.6142	0.3709	0.2190	0.042*	
H10B	0.6106	0.2584	0.2555	0.042*	
N2	0.78665 (12)	0.4798 (3)	0.08874 (16)	0.0258 (7)	
H2A	0.7910	0.5514	0.0931	0.039*	
H2B	0.7694	0.4571	0.1185	0.039*	
H2C	0.7702	0.4638	0.0438	0.039*	
C11	0.83594 (14)	0.4248 (3)	0.10692 (19)	0.0241 (8)	
H11	0.8307	0.3469	0.0971	0.029*	
C12	0.86113 (15)	0.4396 (3)	0.1867 (2)	0.0279 (9)	
H12A	0.8647	0.5166	0.1977	0.034*	
H12B	0.8406	0.4080	0.2146	0.034*	
C13	0.91179 (16)	0.3863 (4)	0.2071 (2)	0.0383 (11)	
H13A	0.9277	0.3997	0.2574	0.046*	
H13B	0.9080	0.3083	0.2002	0.046*	
C14	0.94433 (17)	0.4298 (5)	0.1621 (2)	0.0453 (12)	
H14A	0.9758	0.3915	0.1743	0.054*	
H14B	0.9510	0.5062	0.1726	0.054*	
C15	0.91917 (16)	0.4157 (4)	0.0819 (2)	0.0394 (11)	
H15A	0.9397	0.4481	0.0543	0.047*	
H15B	0.9159	0.3389	0.0705	0.047*	
C16	0.86792 (15)	0.4688 (4)	0.0610 (2)	0.0314 (9)	
H16A	0.8714	0.5469	0.0673	0.038*	
H16B	0.8520	0.4544	0.0108	0.038*	
N3	0.83237 (12)	0.1870 (3)	0.36505 (17)	0.0275 (7)	
H3A	0.8182	0.1415	0.3298	0.041*	
H3B	0.8172	0.2511	0.3573	0.041*	
H3C	0.8299	0.1601	0.4067	0.041*	
C17	0.88578 (15)	0.2008 (3)	0.3675 (2)	0.0291 (9)	
H17	0.8875	0.2434	0.3255	0.035*	
C18	0.91144 (17)	0.2645 (4)	0.4336 (2)	0.0428 (12)	
H18A	0.9080	0.2269	0.4760	0.051*	
H18B	0.8961	0.3355	0.4321	0.051*	
C19	0.96627 (18)	0.2780 (5)	0.4377 (3)	0.0557 (15)	
H19A	0.9697	0.3220	0.3978	0.067*	
H19B	0.9827	0.3155	0.4818	0.067*	
C20	0.9907 (2)	0.1698 (6)	0.4358 (4)	0.081 (2)	
H20A	0.9896	0.1276	0.4777	0.097*	
H20B	1.0252	0.1805	0.4367	0.097*	
C21	0.9641 (2)	0.1080 (5)	0.3677 (5)	0.085 (2)	
H21A	0.9674	0.1479	0.3259	0.103*	
H21B	0.9796	0.0375	0.3676	0.103*	
C22	0.90922 (19)	0.0926 (4)	0.3630 (4)	0.0556 (15)	
H22A	0.8927	0.0574	0.3181	0.067*	
H22B	0.9057	0.0464	0.4020	0.067*	
Cl3	0.76656 (4)	−0.00855 (8)	0.28368 (5)	0.0305 (2)	
O9	0.80156 (11)	0.3999 (2)	0.35850 (16)	0.0352 (7)	
H1O	0.7804	0.4291	0.3195	0.042*	
H2O	0.7911	0.4075	0.3965	0.042*	

### Atomic displacement parameters (Å^2^)

**Table:**

	*U*^11^	*U*^22^	*U*^33^	*U*^12^	*U*^13^	*U*^23^
Sn	0.02068 (13)	0.02730 (13)	0.01990 (12)	−0.00111 (11)	0.00525 (9)	0.00308 (11)
O1	0.0342 (16)	0.0295 (14)	0.0206 (13)	−0.0004 (12)	0.0103 (12)	0.0007 (11)
O2	0.0285 (15)	0.0254 (13)	0.0212 (13)	−0.0018 (11)	0.0086 (11)	0.0002 (11)
O3	0.0387 (18)	0.0303 (15)	0.0258 (15)	0.0031 (13)	0.0102 (13)	−0.0038 (12)
O4	0.0421 (18)	0.0279 (14)	0.0241 (14)	−0.0028 (13)	0.0124 (13)	0.0019 (12)
O5	0.0299 (16)	0.0276 (14)	0.0259 (14)	0.0022 (12)	0.0082 (12)	0.0016 (11)
O6	0.0253 (15)	0.0298 (14)	0.0228 (13)	0.0025 (11)	−0.0007 (11)	−0.0039 (11)
O8	0.0314 (17)	0.0458 (18)	0.0323 (16)	0.0102 (14)	0.0080 (13)	−0.0029 (14)
O7	0.0456 (19)	0.0305 (15)	0.0239 (14)	−0.0047 (13)	0.0102 (13)	−0.0057 (12)
Cl1	0.0225 (5)	0.0539 (7)	0.0497 (7)	0.0053 (5)	0.0107 (5)	0.0141 (5)
Cl2	0.0541 (7)	0.0439 (6)	0.0339 (6)	−0.0059 (5)	0.0175 (5)	0.0134 (5)
C3	0.031 (2)	0.0275 (19)	0.0209 (19)	−0.0055 (16)	0.0071 (17)	0.0012 (16)
C4	0.026 (2)	0.0270 (19)	0.026 (2)	0.0059 (16)	0.0112 (17)	0.0080 (16)
C1	0.023 (2)	0.0279 (19)	0.0219 (19)	0.0056 (16)	0.0062 (16)	0.0025 (15)
C2	0.026 (2)	0.0256 (19)	0.0221 (19)	0.0021 (15)	0.0086 (16)	0.0042 (15)
N1	0.034 (2)	0.0362 (19)	0.0209 (16)	0.0000 (15)	0.0105 (15)	−0.0005 (14)
C5	0.027 (2)	0.034 (2)	0.0196 (19)	0.0017 (17)	0.0045 (16)	0.0037 (16)
C6	0.035 (2)	0.033 (2)	0.032 (2)	−0.0027 (18)	0.0093 (19)	−0.0007 (18)
C7	0.040 (3)	0.051 (3)	0.041 (3)	−0.007 (2)	0.004 (2)	−0.006 (2)
C8	0.026 (2)	0.079 (4)	0.039 (3)	−0.012 (2)	0.002 (2)	−0.003 (3)
C9	0.033 (3)	0.063 (3)	0.046 (3)	0.008 (2)	0.006 (2)	−0.006 (2)
C10	0.032 (2)	0.037 (2)	0.033 (2)	0.0022 (19)	0.0059 (19)	−0.0068 (19)
N2	0.0258 (17)	0.0314 (17)	0.0195 (15)	−0.0007 (14)	0.0048 (13)	−0.0006 (14)
C11	0.023 (2)	0.0265 (19)	0.0206 (18)	0.0025 (15)	0.0024 (15)	0.0011 (15)
C12	0.029 (2)	0.036 (2)	0.0187 (18)	−0.0034 (17)	0.0055 (16)	0.0026 (16)
C13	0.029 (2)	0.057 (3)	0.026 (2)	0.002 (2)	0.0027 (18)	0.009 (2)
C14	0.028 (2)	0.070 (3)	0.035 (2)	0.002 (2)	0.003 (2)	−0.001 (2)
C15	0.025 (2)	0.059 (3)	0.037 (2)	0.008 (2)	0.0132 (19)	−0.001 (2)
C16	0.031 (2)	0.041 (2)	0.0231 (19)	−0.0006 (19)	0.0096 (17)	0.0002 (17)
N3	0.0274 (18)	0.0340 (18)	0.0207 (16)	−0.0031 (14)	0.0057 (14)	−0.0012 (14)
C17	0.024 (2)	0.035 (2)	0.029 (2)	−0.0061 (17)	0.0088 (17)	0.0016 (17)
C18	0.033 (3)	0.060 (3)	0.034 (2)	−0.012 (2)	0.005 (2)	0.000 (2)
C19	0.030 (3)	0.072 (4)	0.057 (3)	−0.020 (3)	−0.001 (2)	0.006 (3)
C20	0.023 (3)	0.084 (5)	0.124 (6)	−0.001 (3)	0.002 (3)	0.039 (5)
C21	0.042 (4)	0.057 (4)	0.169 (8)	0.011 (3)	0.049 (5)	0.002 (5)
C22	0.036 (3)	0.049 (3)	0.085 (4)	0.003 (2)	0.023 (3)	−0.004 (3)
Cl3	0.0300 (5)	0.0338 (5)	0.0303 (5)	−0.0063 (4)	0.0126 (4)	−0.0057 (4)
O9	0.0339 (17)	0.0423 (17)	0.0297 (15)	0.0034 (14)	0.0091 (13)	0.0073 (13)

### Geometric parameters (Å, º)

**Table:**

Sn—O5	2.060 (3)	C11—C16	1.520 (5)
Sn—O6	2.097 (3)	C11—C12	1.526 (5)
Sn—O1	2.097 (3)	C11—H11	0.9800
Sn—O2	2.098 (3)	C12—C13	1.516 (6)
Sn—Cl1	2.3370 (11)	C12—H12A	0.9700
Sn—Cl2	2.3466 (10)	C12—H12B	0.9700
O1—C1	1.306 (5)	C13—C14	1.516 (6)
O2—C2	1.281 (5)	C13—H13A	0.9700
O3—C1	1.206 (5)	C13—H13B	0.9700
O4—C2	1.222 (4)	C14—C15	1.531 (6)
O5—C3	1.303 (5)	C14—H14A	0.9700
O6—C4	1.282 (5)	C14—H14B	0.9700
O8—C4	1.214 (5)	C15—C16	1.529 (6)
O7—C3	1.215 (5)	C15—H15A	0.9700
C3—C4	1.572 (6)	C15—H15B	0.9700
C1—C2	1.561 (5)	C16—H16A	0.9700
N1—C5	1.500 (5)	C16—H16B	0.9700
N1—H1A	0.8900	N3—C17	1.493 (5)
N1—H1B	0.8900	N3—H3A	0.8900
N1—H1C	0.8900	N3—H3B	0.8900
C5—C6	1.513 (6)	N3—H3C	0.8900
C5—C10	1.514 (6)	C17—C22	1.498 (6)
C5—H5	0.9800	C17—C18	1.508 (6)
C6—C7	1.522 (6)	C17—H17	0.9800
C6—H6A	0.9700	C18—C19	1.524 (7)
C6—H6B	0.9700	C18—H18A	0.9700
C7—C8	1.514 (7)	C18—H18B	0.9700
C7—H7A	0.9700	C19—C20	1.503 (9)
C7—H7B	0.9700	C19—H19A	0.9700
C8—C9	1.514 (7)	C19—H19B	0.9700
C8—H8A	0.9700	C20—C21	1.530 (10)
C8—H8B	0.9700	C20—H20A	0.9700
C9—C10	1.525 (6)	C20—H20B	0.9700
C9—H9A	0.9700	C21—C22	1.526 (7)
C9—H9B	0.9700	C21—H21A	0.9700
C10—H10A	0.9700	C21—H21B	0.9700
C10—H10B	0.9700	C22—H22A	0.9700
N2—C11	1.492 (5)	C22—H22B	0.9700
N2—H2A	0.8900	O9—H1O	0.8992
N2—H2B	0.8900	O9—H2O	0.8667
N2—H2C	0.8900		
			
O5—Sn—O6	79.99 (10)	N2—C11—C16	110.6 (3)
O5—Sn—O1	163.31 (11)	N2—C11—C12	109.4 (3)
O6—Sn—O1	87.22 (10)	C16—C11—C12	111.3 (3)
O5—Sn—O2	89.79 (10)	N2—C11—H11	108.5
O6—Sn—O2	84.16 (11)	C16—C11—H11	108.5
O1—Sn—O2	78.19 (10)	C12—C11—H11	108.5
O5—Sn—Cl1	95.71 (8)	C13—C12—C11	111.0 (3)
O6—Sn—Cl1	173.10 (8)	C13—C12—H12A	109.4
O1—Sn—Cl1	95.93 (8)	C11—C12—H12A	109.4
O2—Sn—Cl1	90.46 (8)	C13—C12—H12B	109.4
O5—Sn—Cl2	98.78 (8)	C11—C12—H12B	109.4
O6—Sn—Cl2	88.65 (8)	H12A—C12—H12B	108.0
O1—Sn—Cl2	91.55 (8)	C14—C13—C12	111.2 (4)
O2—Sn—Cl2	167.72 (8)	C14—C13—H13A	109.4
Cl1—Sn—Cl2	97.37 (4)	C12—C13—H13A	109.4
C1—O1—Sn	115.6 (2)	C14—C13—H13B	109.4
C2—O2—Sn	115.3 (2)	C12—C13—H13B	109.4
C3—O5—Sn	114.9 (2)	H13A—C13—H13B	108.0
C4—O6—Sn	114.7 (2)	C13—C14—C15	110.9 (4)
O7—C3—O5	125.1 (4)	C13—C14—H14A	109.5
O7—C3—C4	119.5 (4)	C15—C14—H14A	109.5
O5—C3—C4	115.4 (3)	C13—C14—H14B	109.5
O8—C4—O6	125.7 (4)	C15—C14—H14B	109.5
O8—C4—C3	119.3 (4)	H14A—C14—H14B	108.0
O6—C4—C3	115.0 (3)	C16—C15—C14	111.4 (4)
O3—C1—O1	125.8 (4)	C16—C15—H15A	109.4
O3—C1—C2	120.4 (3)	C14—C15—H15A	109.4
O1—C1—C2	113.9 (3)	C16—C15—H15B	109.4
O4—C2—O2	124.7 (3)	C14—C15—H15B	109.4
O4—C2—C1	119.6 (3)	H15A—C15—H15B	108.0
O2—C2—C1	115.7 (3)	C11—C16—C15	110.5 (3)
C5—N1—H1A	109.5	C11—C16—H16A	109.6
C5—N1—H1B	109.5	C15—C16—H16A	109.6
H1A—N1—H1B	109.5	C11—C16—H16B	109.6
C5—N1—H1C	109.5	C15—C16—H16B	109.6
H1A—N1—H1C	109.5	H16A—C16—H16B	108.1
H1B—N1—H1C	109.5	C17—N3—H3A	109.5
N1—C5—C6	110.8 (3)	C17—N3—H3B	109.5
N1—C5—C10	109.8 (3)	H3A—N3—H3B	109.5
C6—C5—C10	111.5 (4)	C17—N3—H3C	109.5
N1—C5—H5	108.2	H3A—N3—H3C	109.5
C6—C5—H5	108.2	H3B—N3—H3C	109.5
C10—C5—H5	108.2	N3—C17—C22	110.3 (3)
C5—C6—C7	110.4 (4)	N3—C17—C18	109.4 (3)
C5—C6—H6A	109.6	C22—C17—C18	113.3 (4)
C7—C6—H6A	109.6	N3—C17—H17	107.9
C5—C6—H6B	109.6	C22—C17—H17	107.9
C7—C6—H6B	109.6	C18—C17—H17	107.9
H6A—C6—H6B	108.1	C17—C18—C19	110.2 (4)
C8—C7—C6	112.0 (4)	C17—C18—H18A	109.6
C8—C7—H7A	109.2	C19—C18—H18A	109.6
C6—C7—H7A	109.2	C17—C18—H18B	109.6
C8—C7—H7B	109.2	C19—C18—H18B	109.6
C6—C7—H7B	109.2	H18A—C18—H18B	108.1
H7A—C7—H7B	107.9	C20—C19—C18	111.1 (5)
C9—C8—C7	111.0 (4)	C20—C19—H19A	109.4
C9—C8—H8A	109.4	C18—C19—H19A	109.4
C7—C8—H8A	109.4	C20—C19—H19B	109.4
C9—C8—H8B	109.4	C18—C19—H19B	109.4
C7—C8—H8B	109.4	H19A—C19—H19B	108.0
H8A—C8—H8B	108.0	C19—C20—C21	110.1 (5)
C8—C9—C10	110.8 (4)	C19—C20—H20A	109.6
C8—C9—H9A	109.5	C21—C20—H20A	109.6
C10—C9—H9A	109.5	C19—C20—H20B	109.6
C8—C9—H9B	109.5	C21—C20—H20B	109.6
C10—C9—H9B	109.5	H20A—C20—H20B	108.2
H9A—C9—H9B	108.1	C22—C21—C20	111.2 (6)
C5—C10—C9	110.7 (4)	C22—C21—H21A	109.4
C5—C10—H10A	109.5	C20—C21—H21A	109.4
C9—C10—H10A	109.5	C22—C21—H21B	109.4
C5—C10—H10B	109.5	C20—C21—H21B	109.4
C9—C10—H10B	109.5	H21A—C21—H21B	108.0
H10A—C10—H10B	108.1	C17—C22—C21	109.6 (4)
C11—N2—H2A	109.5	C17—C22—H22A	109.8
C11—N2—H2B	109.5	C21—C22—H22A	109.8
H2A—N2—H2B	109.5	C17—C22—H22B	109.8
C11—N2—H2C	109.5	C21—C22—H22B	109.8
H2A—N2—H2C	109.5	H22A—C22—H22B	108.2
H2B—N2—H2C	109.5	H1O—O9—H2O	111.9

### Hydrogen-bond geometry (Å, º)

**Table:**

*D*—H···*A*	*D*—H	H···*A*	*D*···*A*	*D*—H···*A*
N1—H1*A*···O4^i^	0.89	2.11	2.957 (4)	160
N1—H1*B*···Cl3^i^	0.89	2.29	3.163 (4)	166
N1—H1*C*···O8	0.89	2.05	2.873 (4)	154
N1—H1*C*···O7	0.89	2.50	3.130 (5)	129
N2—H2*A*···O4^ii^	0.89	1.99	2.829 (4)	157
N2—H2*A*···O3^ii^	0.89	2.56	3.197 (4)	129
N2—H2*B*···Cl3^i^	0.89	2.41	3.209 (3)	150
N2—H2*C*···O6^iii^	0.89	2.00	2.879 (4)	170
N3—H3*A*···Cl3	0.89	2.37	3.180 (3)	152
N3—H3*A*···O7	0.89	2.48	2.971 (4)	115
N3—H3*B*···O9	0.89	1.88	2.751 (5)	164
N3—H3*C*···O1^iv^	0.89	2.08	2.957 (4)	167
O9—H1*O*···Cl3^i^	0.90	2.21	3.108 (3)	173
O9—H2*O*···O3^iv^	0.87	2.28	2.950 (4)	135

Symmetry codes: (i) −*x*+3/2, *y*+1/2, −*z*+1/2; (ii) *x*, *y*+1, *z*; (iii) −*x*+3/2, −*y*+1/2, −*z*; (iv) *x*, −*y*, *z*+1/2.
